# Improving the Conservation of Mediterranean Chondrichthyans: The ELASMOMED DNA Barcode Reference Library

**DOI:** 10.1371/journal.pone.0170244

**Published:** 2017-01-20

**Authors:** Alessia Cariani, Silvia Messinetti, Alice Ferrari, Marco Arculeo, Juan J. Bonello, Leanne Bonnici, Rita Cannas, Pierluigi Carbonara, Alessandro Cau, Charis Charilaou, Najib El Ouamari, Fabio Fiorentino, Maria Cristina Follesa, Germana Garofalo, Daniel Golani, Ilaria Guarniero, Robert Hanner, Farid Hemida, Omar Kada, Sabrina Lo Brutto, Cecilia Mancusi, Gabriel Morey, Patrick J. Schembri, Fabrizio Serena, Letizia Sion, Marco Stagioni, Angelo Tursi, Nedo Vrgoc, Dirk Steinke, Fausto Tinti

**Affiliations:** 1 Department of Biological, Geological and Environmental Sciences, University of Bologna, Ravenna, Italy; 2 Department STEBICEF, University of Palermo, Palermo, Italy; 3 University of Malta, Msida, Malta; 4 Department of Life and Environmental Sciences, University of Cagliari, Cagliari, Italy; 5 COISPA Technology and Research, Bari, Italy; 6 Department of Fisheries and Marine Research, Ministry of Agriculture, Rural Development and Environment, Nicosia, Republic of Cyprus; 7 Centre Régional de Institut National Recherche Halieutique, Nador, Morocco; 8 Institute for Coastal Marine Environment (IAMC) National Research Council (CNR), Mazara del Vallo, Italy; 9 Department of Evolution, Systematics and Ecology, The Hebrew University of Jerusalem, Jerusalem, Israel; 10 Department DIMEVET, University of Bologna, Ozzano dell'Emilia, Italy; 11 Centre for Biodiversity Genomics, Biodiversity Institute of Ontario, University of Guelph, Guelph, Ontario, Canada; 12 Ecole Nationale Superieure des Sciences de la Mer et d'Amenagement du Littoral, Campus Universitaire de Dely Ibrahim, Alger, Algeria; 13 Regional Agency for Environmental Protection-Toscana (ARPAT), Livorno, Italy; 14 Asociación Ondine, Palma de Mallorca, Spain; 15 Department of Biology, University of Bari Aldo Moro, Bari, Italy; 16 Institute of Oceanography and Fisheries, Split, Croatia; University of California Davis, UNITED STATES

## Abstract

Cartilaginous fish are particularly vulnerable to anthropogenic stressors and environmental change because of their K-selected reproductive strategy. Accurate data from scientific surveys and landings are essential to assess conservation status and to develop robust protection and management plans. Currently available data are often incomplete or incorrect as a result of inaccurate species identifications, due to a high level of morphological stasis, especially among closely related taxa. Moreover, several diagnostic characters clearly visible in adult specimens are less evident in juveniles. Here we present results generated by the ELASMOMED Consortium, a regional network aiming to sample and DNA-barcode the Mediterranean Chondrichthyans with the ultimate goal to provide a comprehensive DNA barcode reference library. This library will support and improve the molecular taxonomy of this group and the effectiveness of management and conservation measures. We successfully barcoded 882 individuals belonging to 42 species (17 sharks, 24 batoids and one chimaera), including four endemic and several threatened ones. Morphological misidentifications were found across most orders, further confirming the need for a comprehensive DNA barcoding library as a valuable tool for the reliable identification of specimens in support of taxonomist who are reviewing current identification keys. Despite low intraspecific variation among their barcode sequences and reduced samples size, five species showed preliminary evidence of phylogeographic structure. Overall, the ELASMOMED initiative further emphasizes the key role accurate DNA barcoding libraries play in establishing reliable diagnostic species specific features in otherwise taxonomically problematic groups for biodiversity management and conservation actions.

## Introduction

To assess and conserve biodiversity, it is critical to correctly identify species that occur in a given ecosystem in order to evaluate species richness and abundance. In recent years, methods of identification based on morphology were gradually integrated with methods based on DNA sequences, such as DNA barcoding thereby forming the so called molecular taxonomy. DNA barcoding seeks to advance both specimens identification and species discovery through the analysis of patterns of sequences divergence of a universal, standardized gene region. Many studies have shown the effectiveness of the mitochondrial cytochrome c oxidase subunit I (COI) gene as a universal barcode sequence for species identification in animal lineages [1,2]. Molecular taxonomy overcomes some problems posed by traditional morphological identification such as homoplasy [3] and phenotypic plasticity [4] of characters used for species identification. Secondly, because often the adopted morphological keys are effective only for a particular life stage, many individuals, especially in their juvenile phases, cannot be assigned to species [5]. Finally, traditional taxonomy doesn’t allow the identification of cryptic species [6]. DNA barcoding has been fundamental in case studies related to immature specimens’ identification (e.g. fish larvae, [5,7]; amphibians and reptiles, [8,9]). In many cases it has been successfully employed to resolve species boundaries between morphologically conserved taxa (e.g. tribe Bombini, [10]).

In the marine realm, the conservation of similar morphological traits appears quite common among sibling species [11–14] and it is often combined with the lack of visual communication in many taxa, in favour of chemical [15] or electrical signals [16–18], both intrinsic conditions to the definition of cryptic species. Among marine organisms, Chondrichthyans seem to have experienced frequent cryptic speciation events across different taxa: large lantern sharks [19], skates [20–22], blacktip sharks [23], hammerhead sharks [24] and guitarfish [25] might have undergone isolating mechanisms which precluded mating between co-occurring species. Despite controversies and criticisms [26,27], cryptic species discovery and, in general, species cataloguing are fundamental, as species represent the basic unit for the management, conservation, and legal protection of biodiversity and for the distribution of limited resources [28–33].

To maximize the potential of molecular taxonomy is necessary to have solid and comprehensive DNA barcodes reference libraries. The Fish Barcode of Life campaign (Fish-BOL, http://www.fishbol.org) is an initiative started in 2005 with the goal to barcode all fish species [34–36]. As of August 2016, 665 out of 1228 chondrichthyan species have been barcoded. Fish-BOL’s efforts comprise of numerous projects covering low-level taxonomic groups and several projects that have targeted specific regional chondrichthyan faunas [37–43]. In addition, recent studies demonstrated the effectiveness of DNA barcodes in describing phylogeographic patterns in this class [22,44,45].

One of those targeted Fish-BOL projects is the ELASMOMED Consortium, a regional network active since 2009 involving fish biologists, fishery scientists, and molecular zoologists from 15 research institutions, most of which also participate in the Mediterranean International Trawl Survey (MEDITS) scientific program[46]. The aims of this network are the sampling and barcoding Chondrichthyans of the Mediterranean Sea.

The Mediterranean Sea is appraised as a global marine biodiversity hotspot and it is home to 89 chondrichthyan species (sharks, skates, rays and chimaeras), which corresponds to about 7% of the global species diversity of the group [47–51]. In detail, the Mediterranean Chondrichthyans include one chimaera, 49 sharks belonging to 17 families and 27 genera, and 39 batoids consisting of nine families with 16 genera. Five batoids are considered endemic: *Leucoraja melitensis*, *Raja asterias*, *Raja radula*, *Raja polystigma*, and *Mobula mobular*, although the endemic status of the latter is uncertain as its separation from the widespread congener *Mobula japonica* has been recently questioned [52]. In parallel, a total of 86 Mediterranean Chondrichthyan species (S1 Table) was reviewed for the European Red List of Marine Fishes [53]. This list includes species threatened by extinction in European waters. Assessed taxa were categorized as Critically Endangered (15), Endangered (13), Vulnerable (11), Near Threatened (15), Least Concern (12) and Data Deficient (20).

Several exotic elasmobranchs have recently been recorded in the Mediterranean (S1 Table), however their particular status (vagrant, alien, or established) is still under debate [48,54–57]. The presence of six species (*Carcharhinus melanopterus*, *Dipturus batis* complex, *Torpedo alexandrinsis*, *Leucoraja fullonica*, *Raja africana*), however, has not been confirmed [50,58–61]. In contrast, the presence of *Dipturus nidarosiensis* in the Mediterranean, a species formerly reported only from the North-Eastern Atlantic, has been ascertained by recent studies [58,62].

Chondrichthyans’ reproductive strategy makes them particularly vulnerable to anthropogenic stressors, such as the use of different fishing gears and the direct and indirect effects of environmental changes and habitat fragmentation [63]. For example, neritic species such as *Squatina* spp. and *Scyliorhinus stellaris* are highly depleted as a consequence of the use of unselective fishing gear [64]. Available fisheries data for Chondrichthyes are incomplete and incorrect, because they are often recorded at higher taxonomic levels than species, with frequent misidentifications of individuals [51,65,66]. In 2012, the General Fisheries Commission for the Mediterranean (GFCM) issued Recommendation GFCM/36/2012/3 on fisheries management measures for conservation of sharks and rays in the GFCM area stating that “cartilaginous fish are kept on board, trans-shipped, landed and marketed at first sale in a way that species are recognizable and identifiable and catches, incidental takings and, whenever appropriate, releases by species can be monitored and recorded” [67]. However, morphological identification of cartilaginous fish remains difficult because of low levels of differentiation among species across multiple taxa and several diagnostic taxonomic characters are clearly exhibited by adult specimens but are less pronounced in juveniles [22,51,65,66]. This can lead to erroneous species attribution even among skate species that are not closely related, e.g. *Leucoraja fullonica* and *L*. *circularis*. Such taxonomic uncertainties often occur within a larger group constituted of *Raja polystigma*, *R*. *montagui*, *R*. *asterias* and *R*. *brachyura* [22,66]. For sharks, similar difficulties were reported for the congeneric species *Squalus blainville* and *Squalus megalops* [68,69] and for *Mustelus mustelus* and *Mustelus punctulatus* [70]. Finally, cryptic species (*sensu* Bickford et al. [6]) have frequently been reported in elasmobranchs as shown by molecular studies of the common skate *Dipturus batis*, once one of the most abundant skate resources in the North-Eastern Atlantic trawl fishery and today heavily depleted in most of its range [63]. *D*. *batis* actually comprises two cryptic species, *Dipturus* cf *intermedia* and *Dipturus* cf *flossada* [20,71,72]. A genetic analysis of Eastern Atlantic and Mediterranean species of the genus *Raja* revealed several recently diverged peripatric sibling species, such as *Raja clavata* and *R*. *straeleni* [73], *R*. *polystigma* and *R*. *montagui* [22,74], as well as the *R*. *miraletus* complex [75,76].

Here we report the establishment of DNA reference barcodes for 42 chondrichthyan species, mostly collected as part of the MEDITS program, as an integrative tool to improve the effectiveness of the above mentioned measures for conservation. This new library will: i) offer a valuable tool for reliable identification of specimens and clarify the taxonomic status of important cartilaginous fishes; ii) support taxonomists who are reviewing the current identification keys, and iii) provide a robust scientific baseline for management and conservation actions, especially in relation to endemic and endangered species. Finally, the comparison and integration of the ELASMOMED dataset with other public barcode datasets will also allow the preliminary identification of geographical population structure and help to determine candidate conservation units.

In general, the assembly of a comprehensive DNA barcode reference library for biological species would mean to compile a biodiversity inventory on different scales and dimensions, making molecular systematics a fundamental tool for the implementation of biodiversity monitoring programmes worldwide. Several of such programmes already exploit the potential of accurate DNA reference libraries by integrating environmental DNA analyses in their standard monitoring programmes [77–79].

## Materials and Methods

### Sampling

Specimens used in this study were collected from Mediterranean individuals caught during scientific research programs. No specific approval of this vertebrate work is required since the individuals sampled in this study were obtained from scientific and commercial fishing activities. A total of 998 individuals were collected from several locations within six Mediterranean FAO fishing divisions (http://www.fao.org/fishery/area/Area37/en). Starting in 2009, dedicated sampling was performed mainly in the framework of the MEDITS scientific surveys (http://www.sibm.it/SITO%20MEDITS/principaleprogramme.htm) or by contracted commercial fishermen on designated cruises (S2 Table). Additional samples were provided by each partner of the ELASMOMED Consortium, from each Institute’s collections. Specimen and collection data, as well as voucher digital images (when recorded) were uploaded to the “ELASMOMED Consortium” project (Project Code: ELAMO) accessible through the Barcode of Life Data system (BOLD, http://www.barcodinglife.org, [80]). Individual fin clips or skeletal muscle tissue samples were collected and preserved in 96% ethanol and kept at -20°C until laboratory analyses.

### DNA extraction, amplification and sequencing

Laboratory work was jointly carried out by the Centre for Biodiversity Genomics (CBG) and at the University of Bologna (UNIBO). At CBG 650bp of the mitochondrial COI region were obtained by following standardized high-throughput protocols for DNA barcode amplification and sequencing [81]. At UNIBO the same COI fragment was amplified using the primer set FishF2 and FishR2 following the protocol described in Ward et al. [82]. Amplification products were checked on a 1.5% agarose gel. A commercial sequence service provider (Macrogen Europe, Amsterdam, Netherlands) performed sequencing employing the same primers used for the amplification. Trace files and sequence data were uploaded to BOLD and subsequently submitted to GenBank (Accession numbers are provided in S3 Table).

### Specimens’ identification and spatial scale of barcode variation

Specimens were identified on board or in the lab using morphological taxonomic characters according to guidelines provided in [83]. A p-distance metric with pairwise deletion was used for sequence comparisons [84]. Genetic distances and Neighbour-joining (NJ) tree clustering [85] were obtained using MEGA version 6 [86]. Confidence in estimated relationships of NJ tree topologies was evaluated by a bootstrap analysis with 1,000 replicates [87].

The mean and maximum intraspecific genetic distances and the mean distance to the Nearest Neighbour (NN) were computed using the ‘Barcoding Gap Analysis’ tool on BOLD [80]. Maximum intraspecific distance was plotted against the mean distance to the NN for each species to infer the presence of a “barcode gap”, which is defined as a distinct gap between intraspecific and interspecific variability [84].

The Barcode Index Number (BIN) System clusters sequences using a Refined Single Linkage algorithm to produce operational taxonomic units that closely correspond to species. BINs were automatically assigned by BOLD and assessed using the ‘BIN Discordance Report’ analysis tool [88]. This tool labels a BIN as “concordant” when it comprises sequences attributed to the same species, and “discordant” when it comprises sequences of different species.

An arbitrary measure of taxonomic reliability was attributed to each barcoded taxon according to the criteria proposed by Costa et al. [89]. Representative barcode sequences for each species were queried using the BOLD Identification Engine with the Species Level option. Grades ranging from A (full concordance) to E (full discordance) were attributed according to the following criteria:

- Grade A- External concordance: unambiguous species match with specimens from other BOLD projects or published sequences. Monophyletic species with a maximum of 2% (patristic) sequence divergence.
- Grade B- Internal concordance: species congruent within our dataset, where at least 3 specimens of the same species are available, with a maximum of 2% (patristic) sequence divergence. No matching sequences found through the BOLD-IDS.
- Grade C- Sub-optimal concordance (possible within species genetic structure): at least 3 specimens of the same species are available within the library and form a monophyletic cluster; however intraspecific distance is greater than 2%; and/or the BOLD-IDS indicates monophyletic nearest neighbour of the same species, with more than 2% patristic distance.
- Grade D- Insufficient Data: low number of specimens analysed (1 or 2 individuals) and no matching sequence available in BOLD.
- Grade E- Discordant species assignments: sequences for a given species in our dataset did not match with the same species in BOLD. The specimen may match with a different species or may display paraphyly or polyphyly.

Because of the several Mediterranean geographical areas covered by ELASMOMED, we tested for the presence of phylogeographic signal at regional level for species with barcode data from multiple FAO divisions. Species-specific haplotype networks were created using Haploviewer (http://www.cibiv.at/~greg/haploviewer). Parsimony trees required for Haploviewer were reconstructed with the *dnapars* of the PHYLIP package version 3.6 [90,91].

## Results

DNA barcodes could be recovered for 884 of the 998 individuals. Stop codons were recorded only for two specimens: ELAMO028-15 (*S*. *blainville*) and ELAME1143-11 (*Torpedo marmorata*), which were excluded from further analyses. The newly generated Mediterranean barcode library ELASMOMED includes 42 species: 17 sharks, 24 skates/rays and one chimera, belonging to eight orders and 18 families (S2 Table). Overall nucleotide frequencies were 25.16% adenine (A), 26.12% cytosine (C), 16.78% guanine (G) and 31.94% thymine (T), with an average GC content of 42.90%.

A first Neighbour-Joining tree of 882 barcode sequences showed that 77 specimens (8.73%), representing 11 species, clustered with individuals of a closely related and morphologically similar species (Table 1), probably because of identification errors during field sampling. All these specimens were reassessed and subsequently renamed.

**Table 1:** Cases of specimens’ misidentification by FAO fishing division. The number of misidentified individuals over the total number of barcoded individuals is given in parenthesis.

Barcode ID	Morphological ID	FAO Division
*Dasyatis centroura*	*Pteroplatytrygon violacea*	37.1.3—Sardina (1/1)
*Pteroplatytrygon violacea*	*Dasyatis centroura*	37.1.1—Balearic (1/1)
*Leucoraja circularis*	*Leucoraja fullonica*	37.1.3—Sardinia (2/8); 37.2.2—Ionian (1/5)
*Raja polystigma*	*Raja montagui*	37.1.1—Balearic (2/5)
*Torpedo marmorata*	*Torpedo nobiliana*	37.3.2—Levant (3/9)
*Torpedo marmorata*	*Torpedo torpedo*	37.2.1—Adriatic (1/1)
*Scyliorhinus canicula*	*Scyliorhinus stellaris*	37.2.1—Adriatic (4/20)
*Mustelus punctulatus*	*Mustelus mustelus*	37.1.1—Balearic (5/5); 37.2.1—Adriatic (45/146)
*Centrophorus granulosus*	*Centrophorus uyato*	37.1.1—Balearic (1/5)
*Squalus blainville*	*Squalus acanthias*	37.1.1—Balearic (2/3); 37.3.2—Levant (5/5)
*Squalus blainville*	*Squalus megalops*	37.2.2—Ionian (4/12)

**Fig 1:**
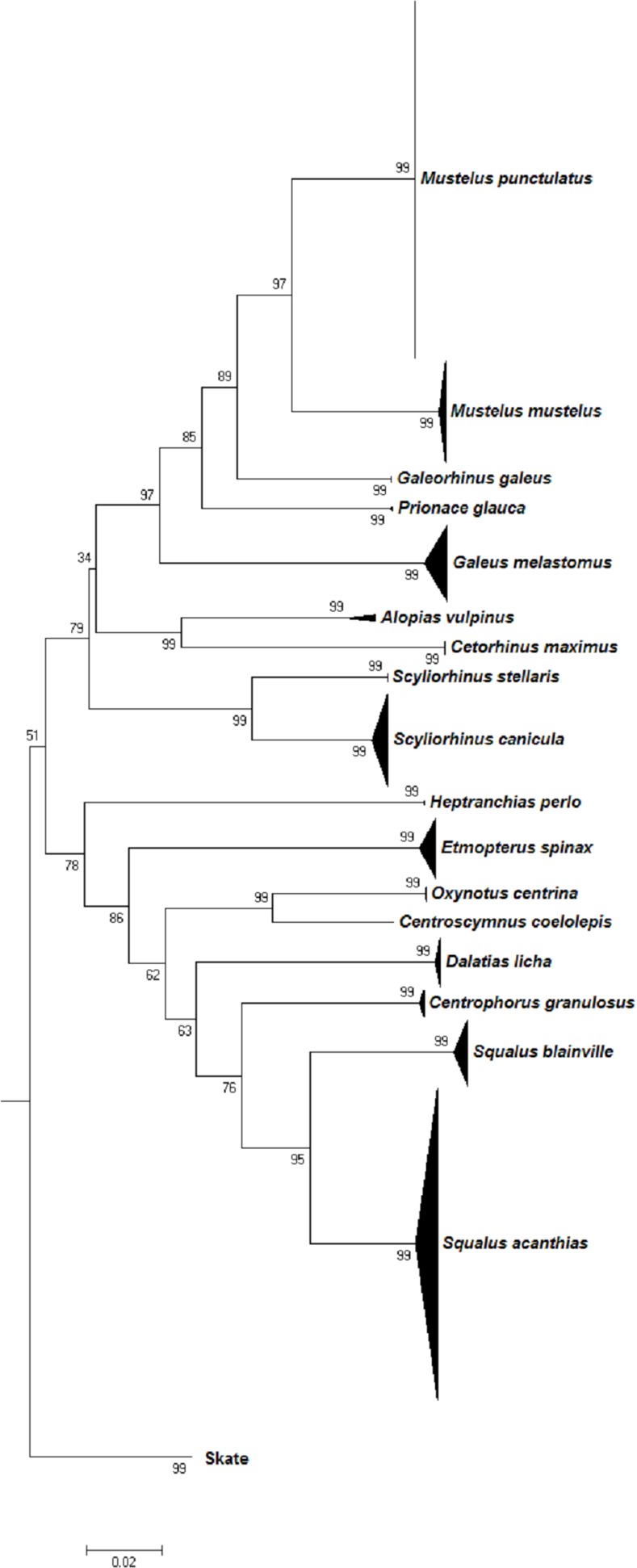
Neighbour-Joining tree based on genetic p-distances of COI barcode sequences of 17 shark species. Numbers near nodes indicate bootstrap values (>50%). The distance scale bar is given. Each species' sample size and geographic origin are detailed in S2 Table.

**Fig 2:**
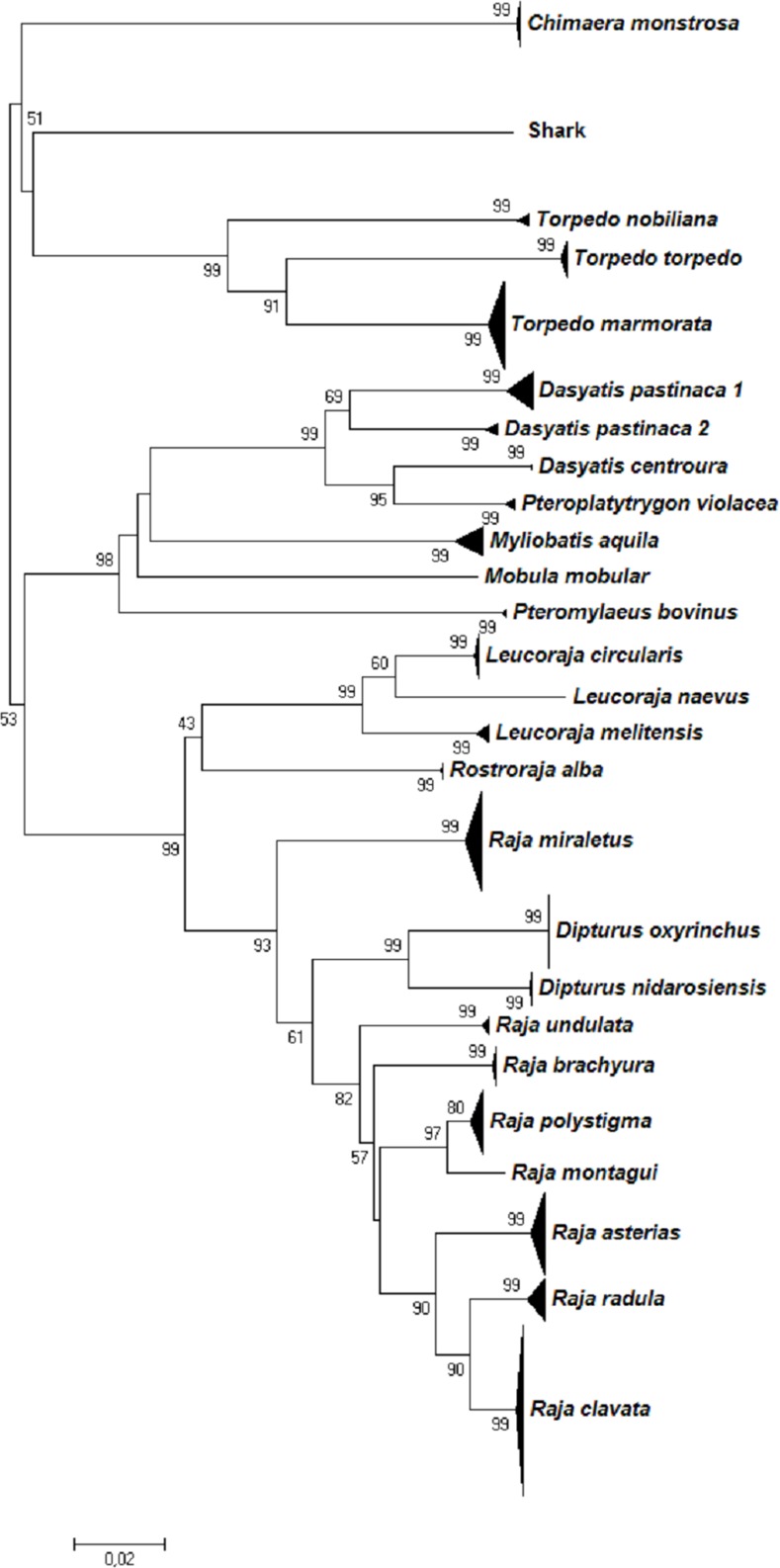
Neighbour-Joining tree based on genetic p-distances of COI barcode sequences of 23 skate species. Numbers near nodes indicate bootstrap values (>50%). The distance scale bar is given. Each species' sample size and geographic origin are detailed in S2 Table.

All further analyses were conducted using this curated and corrected dataset and the resulting Neighbour-Joining tree (Figs 1 and 2) indicated that most of the species formed cohesive units, concordant with the morphological identification. Species-specific clusters were supported by high bootstrap values (≥80), with the exception of the *D*. *pastinaca* cluster, which split into two fully supported sub-clusters (1 and 2; Fig 2, S1 Fig). The families Scyliorhinidae and Myliobatidae did not form a monophyletic clade, as did the genera *Raja* and *Dasyatis* (Figs 1 and 2).

**Fig 3:**
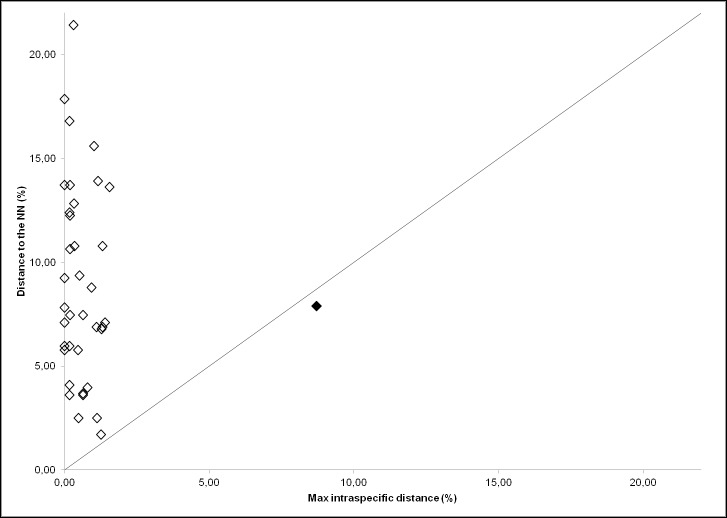
Maximum intraspecific distance plotted against Nearest Neighbour distance (p-distance values) for the COI barcode sequences of the 41 chondrichthyan species. The black rhombus represents the value for *Dasyatis pastinaca* before its separation into two sub-clusters 1 and 2. The 1:1 equivalence (straight line) is indicated.

The mean and maximum p-distance within species showed average values of 0.29% (range = 0–3.06%) and 0.93% (range = 0–8.72%), respectively (Table 2). The highest values for both distances occurred in *Dasyatis pastinaca*. Excluding this outlier taxon, highest mean and maximum p-distances were reduced to 0.43% (*Raja asterias*) and 1.55% (*Galeus melastomus*), respectively. The distances to the Nearest Neighbour (Table 2) varied from 1.71% (*R*. *montagui* vs. *R*. *polystigma*) to 12.83% (*T*. *marmorata* vs. *Torpedo nobiliana*) for congeneric taxa; while higher values were observed for comparisons at higher taxonomic levels (Table 2). Note that the maximum value of intraspecific p-distance was always lower than the distance to the Nearest Neighbour with the exception of *D*. *pastinaca*, highlighting the absence of a “barcode gap” for this species (Fig 3).

**Table 2:** Mean and maximum intraspecific distances and Nearest-Neighbour distance (p-distance values) of the 41 chondrichthyan species barcoded for the ELASMOMED library. The *Dasyatis pastinaca* cluster is not separated.

Species	Within-species distance		Nearest Neighbour	Distance to theNearest Neighbor
	Mean	Maximum		
*Chimaera monstrosa*	0.08	0.31	*Raja polystigma*	21.42
*Dasyatis centroura*	0	0	*Pteroplatytrygon violacea*	5.77
*Dasyatis pastinaca*	3.06	8.72	*Pteroplatytrygon violacea*	7.89
*Pteroplatytrygon violacea*	0.24	0.46	*Dasyatis centroura*	5.77
*Mobula mobular*	N/A	N/A	*Myliobatis aquila*	16.36
*Myliobatis aquila*	0.29	1.02	*Pteroplatytrygon violacea*	15.59
*Pteromylaeus bovinus*	0.11	0.17	*Myliobatis aquila*	16.79
*Dipturus nidarosiensis*	0.03	0.16	*Dipturus oxyrinchus*	5.96
*Dipturus oxyrinchus*	0	0	*Dipturus nidarosiensis*	5.96
*Leucoraja circularis*	0.03	0.16	*Leucoraja melitensis*	3.60
*Leucoraja melitensis*	0.34	0.63	*Leucoraja circularis*	3.60
*Leucoraja naevus*	N/A	N/A	*Leucoraja circularis*	5.02
*Raja asterias*	0.43	0.79	*Raja radula*	3.96
*Raja brachyura*	0.08	0.16	*Raja polystigma*	4.09
*Raja clavata*	0.06	0.47	*Raja radula*	2.51
*Raja miraletus*	0.30	1.28	*Raja polystigma*	6.79
*Raja montagui*	N/A	N/A	*Raja polystigma*	1.71
*Raja polystigma*	0.31	1.26	*Raja montagui*	1.71
*Raja radula*	0.35	1.11	*Raja clavata*	2.51
*Raja undulata*	0.31	0.64	*Raja polystigma*	3.68
*Rostroraja alba*	0.05	0.18	*Leucoraja melitensis*	12.24
*Torpedo marmorata*	0.42	1.30	*Torpedo torpedo*	10.77
*Torpedo nobiliana*	0.19	0.33	*Torpedo marmorata*	12.83
*Torpedo torpedo*	0.05	0.34	*Torpedo marmorata*	10.77
*Prionace glauca*	0.19	0.19	*Galeorhinus galeus*	10.63
*Galeus melastomus*	0.24	1.55	*Galeorhinus galeus*	13.63
*Scyliorhinus canicula*	0.35	1.40	*Scyliorhinus stellaris*	7.10
*Scyliorhinus stellaris*	0	0	*Scyliorhinus canicula*	7.10
*Galeorhinus galeus*	0	0	*Mustelus punctulatus*	9.25
*Mustelus mustelus*	0.07	0.64	*Mustelus punctulatus*	7.46
*Mustelus punctulatus*	0	0.18	*Mustelus mustelus*	7.46
*Heptranchias perlo*	0	0	*Centroscymnus coelolepis*	17.85
*Alopias vulpinus*	0.12	0.18	*Cetorhinus maximus*	13.72
*Cetorhinus maximus*	0	0	*Alopias vulpinus*	13.72
*Centrophorus granulosus*	0.08	0.52	*Squalus acanthias*	9.36
*Dalatias licha*	0.05	0.16	*Squalus acanthias*	12.40
*Etmopterus spinax*	0.28	1.15	*Centrophorus granulosus*	13.91
*Oxynotus centrina*	0	0	*Centroscymnus coelolepis*	7.82
*Centroscymnus coelolepis*	N/A	N/A	*Oxynotus centrina*	7.82
*Squalus acanthias*	0.11	1.10	*Squalus blainville*	6.87
*Squalus blainville*	0.33	1.30	*Squalus acanthias*	6.87
Average	0.29	0.93		8.79

The BIN discordance analysis showed 17 discordant BINs out of 42 (40.47%). After reviewing the reasons for the observed discordance, 11 BINs could be reclassified as concordant (Table 3). This included cases such as the *M*. *mustelus* BIN with 72 records of *M*. *mustelus* and three sequences attributed to a provisional name, indicating that the discordance was caused by the use of an interim name. Other cases could be ascribed to erroneous morphological identifications of one or two records associated with the BIN, e.g. *Raja clavata*, where a few records were assigned differently. The five remaining discordant BINs contained several individuals belonging to more than one species, indicating that for these species either the barcode sequence or the BIN algorithm is not sufficient to discriminate them. However, the possibility of misidentification cannot be excluded either. Lastly, BIN AAD5036 associated with the *D*. *pastinaca* sub-cluster 2 consists of six individuals of *Dasyatis tortonesei* from the Muséum National d’Historie Naturelle, Paris, indicating a possible species misidentification of the four ELASMOMED specimens (Table 3).

**Table 3:** Species with discordant BINs found by the ‘BIN Discordance Report’ sequence analysis tool on BOLD. Taxonomic ranks of conflict are reported including species of the same genus up to taxa belonging to different orders. The column BIN Taxon Variation shows the number of records for each taxon in parenthesis.

Species	Discordant BIN	Rank of conflict	BIN Taxon Variation	After review
*Dasyatis pastinaca 1*	BOLD:ACK8259	Family	Dasyatidae[20], Rhinobatidae[1]	Concordant
*Squalus blainville*	BOLD:AAA1550	Family	Squalidae[131], Centrophoridae[1]	Concordant
*Prionace glauca*	BOLD:AAA7096	Genus	Prionace[68], Carcharhinus[1]	Concordant
*Dasyatis centroura*	BOLD:AAD5044	Species	*Dasyatis ushiei[7]*, *Dasyatis centroura[7]*	**Discordant**
*Dasyatis pastinaca 2*	BOLD:AAD5036	Species	*Dasyatis tortonesei[6]*, *Dasyatis pastinaca[4]*	**Discordant**
*Mobula mobular*	BOLD:AAB8636	Species	*Mobula japanica[27]*, *Mobula mobular[2]*, *Mobula sp*.*[1]*, *Mobula japonica[1]*	**Discordant**
*Dipturus oxyrinchus*	BOLD:ABZ4263	Species	*Dipturus oxyrinchus sp1[56]*, *Dipturus oxyrinchus[49]*	Concordant
*Raja brachyura*	BOLD:AAA4358	Species	*Raja brachyura[36]*, *Raja sp*.*[1]*	Concordant
*Raja clavata*	BOLD:ACF2419	Species	*Raja clavata[107]*, *Raja undulata[1]*	Concordant
*Raja polystigma*	BOLD:ABY6158	Species	*Raja polystigma[24]*, *Raja sp*.*[1]*	Concordant
*Torpedo nobiliana*	BOLD:AAC6970	Species	*Torpedo nobiliana[9]*, *Torpedo macneilli[5]*, *Torpedo fairchildi[4]*, *Torpedo tokionis[1]*, *Torpedo sp*. *A[1]*	**Discordant**
*Mustelus mustelus*	BOLD:AAA4345	Species	*Mustelus mustelus[72]*, *Mustelus sp*.*[3]*	Concordant
*Mustelus punctulatus*	BOLD:AAA4347	Species	*Mustelus punctulatus[164]*, *Mustelus sp*. *zpl 00058[1]*	Concordant
*Centrophorus granulosus*	BOLD:AAB4327	Species	*Centrophorus granulosus[59]*, *Centrophorus zeehaani[28]*, *Centrophorus lusitanicus[1]*	**Discordant**
*Oxynotus centrina*	BOLD:AAF2493	Species	*Oxynotus centrina[8]*, *Oxynotus paradoxus[1]*	Concordant
*Centroscymnus coelolepis*	BOLD:AAB8284	Species	*Centroscymnus coelolepis[20]*, *Centroscymnus sp*.*[1]*	Concordant
*Squalus acanthias*	BOLD:AAA1547	Species	*Squalus acanthias[242]*, *Squalus suckleyi[32]*	**Discordant**

We applied a ranking system analysis to all the barcoded species, treating the subclusters 1 and 2 of *D*. *pastinaca* as different species. The analysis ranked 36 species at Grade A (85.72%) and six species at Grade E (14.28%) (Table 4), with the last showing the post-revision BIN discordance. As all prior analyses confirmed the misidentification of specimens in the *D*. *pastinaca* sub-cluster 2, the four specimens belonging to this group were assigned to *D*. *tortonesei*, upgrading this cluster to Grade A. Both species are differentiated by 26 nucleotide substitutions (3.66%; S1 Fig). Individuals of *D*. *tortonesei* were found only in the Ionian division (FAO 37.2.2). All four samples were collected in the Strait of Sicily (South-Eastern part of the Ionian FAO division), while those of *D*. *pastinaca* were collected from the divisions Balearic (FAO 37.1.1), Sardinia (FAO 37.1.2) and Ionian (FAO 37.2.2).

**Table 4:** Attribution of grades from A (full concordance) to E (full discordance) to DNA barcodes of the 42 chondrichthyan species of the ELASMOMED reference library, following the ranking system proposed by Costa et al. (2012). The *Dasyatis pastinaca* sub-clusters 1 and 2 are graded separately.

Species	Grade	Species	Grade	Species	Grade
*Chimaera monstrosa*	A	*Raja brachyura*	A	*Scyliorhinus stellaris*	A
*Dasyatis centroura*	E	*Raja clavata*	A	*Galeorhinus galeus*	A
*Dasyatis pastinaca 1*	A	*Raja miraletus*	A	*Mustelus mustelus*	A
*Dasyatis pastinaca 2*	E	*Raja montagui*	A	*Mustelus punctulatus*	A
*Pteroplatytrygon violacea*	A	*Raja polystigma*	A	*Heptranchias perlo*	A
*Mobula mobular*	E	*Raja radula*	A	*Alopias vulpinus*	A
*Myliobatis aquila*	A	*Raja undulata*	A	*Cetorhinus maximus*	A
*Pteromylaeus bovinus*	A	*Rostroraja alba*	A	*Centrophorus granulosus*	E
*Dipturus nidarosiensis*	A	*Torpedo marmorata*	A	*Dalatias licha*	A
*Dipturus oxyrinchus*	A	*Torpedo nobiliana*	E	*Etmopterus spinax*	A
*Leucoraja circularis*	A	*Torpedo torpedo*	A	*Oxynotus centrina*	A
*Leucoraja melitensis*	A	*Prionace glauca*	A	*Centroscymnus coelolepis*	A
*Leucoraja naevus*	A	*Galeus melastomus*	A	*Squalus acanthias*	E
*Raja asterias*	A	*Scyliorhinus canicula*	A	*Squalus blainville*	A

Among juvenile specimens, cases of species misidentification were encountered among smooth hound sharks, cat sharks and dogfish. Misidentification of skates and electric rays were recorded both for juveniles and adults (Table 1). Five juveniles of *M*. *punctulatus* from the Balearic division (FAO 37.1.1) as well as 45 juvenile specimens from the Adriatic division (FAO 37.2.1) were misidentified as *M*. *mustelus*, confirming that the characters used in the taxonomic keys are not adequate for immature individuals. The congeneric species *Scyliorhinus canicula* and *S*. *stellaris* collected in the Adriatic division (FAO 37.2.1) were confused in four cases. Moreover, four out of the 12 dogfishes collected in the Ionian (Strait of Sicily) division (FAO 37.2.2) were initially assigned morphologically to *S*. *megalops*, but were barcoded as *S*. *blainville*. A similar taxonomic confusion between *Squalus acanthias* and *S*. *blainville* occurred in both Balearic (FAO 37.1.1) and Levant (FAO 37.3.2) divisions.

Among rays and skates, *T*. *marmorata* individuals, one from Adriatic (FAO 37.2.1) and three from Levant (FAO 37.3.2) divisions were morphologically misidentified as *T*. *nobiliana* or *T*. *torpedo* because diagnostic spots are absent in juvenile specimens (Table 1). Two specimens of *R*. *polystigma* were misidentified as *R*. *montagui*. Both were collected in the Balearic division (FAO 37.1.1), a unique Mediterranean area where both species co-occur in sympatry. Other minor morphological misidentifications concern a few specimens of *Centrophorus granulosus*, *Pteroplatytrygon violacea*, *D*. *centroura* and *L*. *circularis* (Table 1).

**Fig 4:**
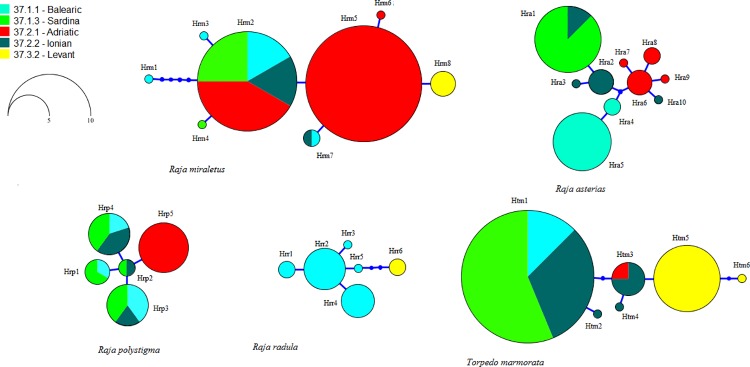
Haplotype parsimony networks of *Raja miraletus*, *Raja asterias*, *Raja polystigma*, *Raja radula* and *Torpedo marmorata* reconstructed using ELASMOMED COI barcode sequences. Each circle represents one haplotype and its size is proportional to frequency. Colours indicate the origin of samples according to FAO fishing divisions. Mutations and positions characterizing each haplotype, as well as the size (bp), and the number of the sequences included in each species network, are provided in S4 Table.

Thirty-four out of the 42 barcoded species were assessed for spatial variation as multiple records were obtained from several FAO divisions (S2 Table). Among those, five batoids showed noticeable phylogeographic signals in the COI sequence variation, mainly represented by private haplotypes of some FAO divisions (Fig 4; the complete list of mutations and positions characterizing each haplotype is reported in S4 Table). Even if the present sample size is too limited to allow solid and definitive phylogeographic inferences, the data allow some preliminary insights from the patterns observed (Fig 4).

*Raja miraletus* showed eight COI haplotypes, with a single private haplotype (Hrm8) comprising all samples from the Levant division. *Raja asterias* consisted of three slightly different haplotype groups: Hra4 and Hra5 for the Balearic division, Hra1-3 for Sardinia and Ionian divisions, and Hra6-9 from the Adriatic division. The latter group also included haplotype Hra10 that contained only a single specimen from the Strait of Sicily. *Raja polystigma* exhibited five haplotypes with the private haplotype Hrp5 of the Adriatic division, while all the others are shared among areas of the Western and Central Mediterranean. Two specimens of *Raja radula* from the Levant division formed private haplotype Hrr6, differentiated by three mutations from the Balearic haplotypes (Hrr1-5). *Torpedo marmorata* contained three haplotype groups: one formed by Htm1 and Htm2 found in the Balearic, Sardinia and Ionian divisions, the second formed by Htm3 and Htm4 found in individuals from Ionian and Adriatic divisions, and the third included the haplotypes Htm5 and Htm6 found only in the Levantine individuals (Fig 4).

## Discussion

### Towards completion of a DNA barcode reference library of the mediterranean chondrichthyes

The ELASMOMED initiative sampled and barcoded 42 out of the 89 chondrichthyan species occurring in the Mediterranean Sea, contributing *ex novo* barcodes for 24 species. In addition, it added several barcoded specimens from different geographical areas to those from previous initiatives. The integration of our results with those of other recent major fish barcoding initiatives carried out in the Mediterranean [39,92] raises the total count to 51 species with DNA barcodes (S1 Table), comprising the holocephalan *Chimaera monstrosa*, 26 sharks (53%), and 24 skates and rays (62%).

As the main source of specimens for the ELASMOMED initiative was the MEDITS program, demersal and bathydemersal species (N = 32) outnumbered pelagic and benthopelagic ones (N = 10; S1 Table). Regarding endemic species, barcode sequences of *R*. *asterias*, *R*. *polystigma* and *M*. *mobular* were newly obtained and the number of records for *L*. *melitensis* and *R*. *radula* greatly increased. As a symptom of the dangerously limited knowledge characterising the Mediterranean chondrichthyans faunas, only one sequence for each species was available until this study [92]. Multiple sequences from each species are needed to properly assess intra- vs inter-specific sequences variation and thus improve the robustness of the molecular assignment. Only one out of nine known alien species (*C*. *altimus*) was barcoded [39]. Of the 38 Mediterranean species without a barcode, 32 have been barcoded from other regions and only six have not been barcoded at all (*Rhinoptera marginata*, *Glaucostegus halavi*, *Pristis pristis*, *Rhinobatos cemiculus*, *Torpedo alexandrinsis* and *Raja africana*; S1 Table).

The ELASMOMED network allowed for an unprecedented large spatial coverage of data encompassing five FAO fishing divisions (S2 Table).Notably, ELASMOMED increased the number of barcoded species from North African countries, as 25 species collected from the Algerian waters were added to those of an earlier study [39]. About 43% of the Mediterranean Chondrichthyan diversity remains to be inventoried and ongoing efforts are needed to increase the number of species collected from both already targeted and remaining areas.

Despite a slower mutation rate of mtDNA genes in sharks [93], our results showed that COI barcoding is a reliable and efficient method for specimens identification, consistent with the findings of previous studies in marine fish [82,89,94,95]. The effectiveness of DNA barcoding was also demonstrated by the cohesive monophyletic clustering in our Neighbour-Joining analysis, the high number of concordant BINs and the majority of Grade A rankings (37 out of 42 species—88.1%) following the Costa *el al*. [89] criteria. Only in five species, categorized as Grade E, barcode sequences did not allow the discrimination of Mediterranean species from congeners inhabiting the Indo-Pacific Ocean, namely *C*. *granulosus* from *Centrophorus zeehaani*, *D*. *centroura* from *Dasyatis ushiei*, *M*. *mobular* from *M*. *japanica*, *S*. *acanthias* from *Squalus suckley* and *T*. *nobiliana* from *Torpedo macneilli*, *Torpedo fairchildi* and *Torpedo tokionis*. This barcode sharing could be attributed to various reasons, such as incomplete lineage sorting, past lineage introgression, taxonomic uncertainties, or matched sequences on BOLD were generated from misidentified specimens.

Although Neighbour-Joining trees based on COI sequence divergence alone are not useful as a phylogenetic tool, they allow limited insights into relationships at higher taxonomic levels [82]. All skate orders and two shark orders (Lamniformes, Squaliformes) were resolved as monophyletic groups with high bootstrap support (98–100)). Intermediate taxonomic levels such as family and genus were not well resolved. For example, the blackmouth catshark, *Galeus melastomus* (Scyliorhinidae) appears closer related to *Prionace glauca* (Carcharhinidae), *M*. *mustelus*, *M*. *punctulatus* and *Galeorhinus galeus* (Triakidae) than to other scyliorhinid sharks such as *S*. *canicula* and *S*. *stellaris;* Myliobatidae (*Myliobatis aquila* and *Pteromylaeus bovinus*) did not form a monophyletic clade, but rather cluster with the whiptail stingrays of the family Dasyatidae. Paraphyletic clusters were also observed for some *Raja*, *Dasyatis* and *Dipturus* species. These data emphasize the need for a classification revision and illustrate the limits of a single marker gene as phylogenetic tool [82]. However, some clusters might reflect insufficient taxonomy, e.g. the placement of *D*. *centroura* close to *P*. *violacea* as opposed to *D*. *pastinaca* and *D*. *tortonesei*, which supports the need for a taxonomic revision of the Mediterranean Dasyatidae. Such unresolved cases could be clarified by using a combined morphological and molecular phylogenetic analyses, as successfully performed for the Indo-Pacific stingrays [96]. The barcode-referenced library of voucher tissues held by ELASMOMED represents a unique opportunity for subsequent research intended to achieve taxonomic improvements.

### From molecular taxonomy to conservation and management issues: the added value of DNA barcoding for chondrichthyans

Persistent problems regarding the morphological taxonomy of elasmobranchs are their pronounced morphological stasis and the fact that distinctive diagnostic characters are only exhibited in adults [51,65,66]. On-board identification of specimens during international trawl surveys usually involves several fisheries scientists from different research institutes or organizations, with varying levels of taxonomic skills and expertise. Field sorting and taxonomic classification are generally carried out using dichotomic keys and identification guides based on characters shown mainly in the adult stage [5]. This approach itself was shown to be misleading, because most individuals caught are either juveniles or sub-adults [83]. The approach adopted by the ELASMOMED Consortium has proven to be effective in detecting cases of misidentification among gulper sharks, smooth hound sharks, cat sharks, sting rays and electric rays. These instances are ascribable to a lack of clear traits and diagnostic characters, especially in immature individuals. The discrimination between *Centrophorus granulosus* and *C*. *uyato* is challenging and the taxonomy of the genus has been controversial and in need of revision[97–99]. The absence of characteristic dorsal black dots on the skin of juveniles of *M*. *punctulatus* (TL < 60cm) can cause confusion with *M*. *mustelus* [70]. Similarly, the characteristic dorsal coloration of mottled light on dark background and the knob-rimmed margins of the spiracles of *Torpedo marmorata* are not pronounced enough in small-size individuals which are often confused with the closely related species *T*. *nobiliana*. In cat sharks, the traits for discriminating *S*. *canicula* and *S*. *stellaris* are the distinct shape of the anterior nasal flap and the distribution pattern of coloured spots on the animal’s skin [39,100]. These diagnostic features were not clearly assessed in a few individuals collected in the Adriatic Sea. The genus *Raja* and, in particular, the sibling sister species *R*. *montagui* and *R*. *polystigma*, are prime examples of taxa with very similar morphological characters both in immature and mature individuals because of a very high level of stasis [22,73,101,102]. Such groups require very accurate, detailed, and time-consuming traditional taxonomic analyses of several individuals in order to assess species-specific characters. DNA barcoding can assign individuals to species even if this is not possible using morphological characters (e.g. in sibling and cryptic species) providing a basic yet very efficient tool to establish reliable diagnostic species-specific features in such problematic taxa. The prerequisite of such an important process is the development and implementation of large, almost complete and constantly updated barcode reference libraries with associated voucher specimens as started by ELASMOMED and other similar studies (e.g. [92]).

Such large repositories consisting of tissues and barcode sequences of dozens of samples per species collected across its entire known distribution area would also allow preliminary exploration of population structure. Intraspecific variation of barcode sequences is intrinsically low, but despite this and the rather low number of individuals and samples analysed we were able to detect signals of phylogeographic structure within some groups of the ELASMOMED Mediterranean dataset. Such preliminary evidence is insufficient and requires further inference of evolutionary patterns suitable for addressing management and conservation issues. The five batoid species *R*. *miraletus*, *R*. *asterias*, *R*. *polystigma*, *R*. *radula* and *T*. *marmorata* exhibited small but detectable phylogeographic structure among the Mediterranean samples, with divergent private haplotypes detected in the Levant area or, whenever the species do not occur that far to east (i.e. *R*. *asterias* and *R*. *polystigma*), in the Adriatic Sea. In addition, *R*. *asterias* showed also two private haplotypes in the Balearic area, likely highlighting a more marked geographical structure than the other four species. Geographical genetic breaks need to be accurately accounted for when delineating management and conservation units. Behavioural ecology and life-history traits of demersal and bathydemersal skates and rays [103–105] make this group particularly prone to genetic structuring and several cases have already been documented for the region [73,106,107]. Other studies used different mtDNA markers such as parts of the control region or cytochrome b (see for *R*. *clavata* [73,106]) to accurately unravel geographical structuring of populations. Recent studies in the Mediterranean used barcode data to assess genetic differentiation of the small-spotted catshark [44,45] and their findings describe phylogeographic signals comparable to those inferred by our ELASMOMED dataset.

Efforts toward the completion of the ELASMOMED database should be continued to expand taxonomic coverage and to improve the assessment of spatial and temporal patterns of species diversity of Chondrichthyes in the Mediterranean. The completion of the ELASMOMED barcode library will allow i) more accurate species-specific information from scientific survey and landing data essential to correctly assess stock status of Chondrichthyans [63,108]; ii) the detection of cryptic and invasive species and iii) the forensic traceability of cartilaginous fish products to fight illegal and unreported fisheries and support commercial trade [109].

DNA barcoding is also the starting point for other fast-evolving DNA-based techniques. The widespread use of environmental DNA allows for the extraction short sequences of multiple-species from complex matrices (e.g. seawater) in form of metabarcoding data [77,110–113].Because of the nature of this methodology, comprehensive barcoding libraries are even more essential in providing valid comparative data and strengthening biodiversity monitoring different geographical scales.

## Supporting Information

S1 Table Summary of all barcoded Mediterranean Chondrichthyans generated by the integration of data from the three main barcoding initiatives by Moftah *et al*. (2011), Landi *et al*. (2014) and ELASMOMED, as well as other available studies (reported under the column OTHER). Both the number of barcode records obtained from Mediterranean and non-Mediterranean specimens are reported. When the number of non-Mediterranean barcodes on BOLD was larger than 10, the exact number is not reported. Species author, Ecology (Bathydemersal, Demersal, Pelagic and Benthopelagic), IUCN Red List Category and Criteria (CR = Critically Endangered, EN = Endangered, VU = Vulnerable, NT = Near Threatened, LC = Least Concern, DD = Data Deficient, NE = Not Evaluated), Status and BIN available on BOLD are detailed for each species. Asterisks denote barcode sequences deposited in public repositories but not in BOLD.
(XLSX)

S2 Table Summary of taxa and individuals collected and barcoded for the ELASMOMED initiative. The number of collected and barcoded individuals are reported for each chondrichthyan species and each FAO division as well as in total.
(XLSX)

S3 Table The GenBank Accession Numbers, BIN, Sample ID and Process ID of the 882 Mediterranean chondrichthyan individuals successfully barcoded by ELASMOMED. (XLSX)

S4 Table Complete list of mutations and positions characterizing each haplotype displayed in Fig 4. For each species, the number of sequences used and the length of the alignment are reported.
(XLSX)

S1 Fig Haplotype parsimony networks of *Dasyatis pastinaca* reconstructed using ELASMOMED COI barcode sequences. Each circle represents one haplotype and its size is proportional to frequency. Colours indicate the origin of samples according to FAO fishing divisions.
(TIF)
