# Supplementary figures and images for: Improving the Conservation of Mediterranean Chondrichthyans: The ELASMOMED DNA Barcode Reference Library
Source: PLoS One. 2017 Jan 20;12(1):e0170244. doi: 10.1371/journal.pone.0170244 (PMC5249125; doi:10.1371/journal.pone.0170244)

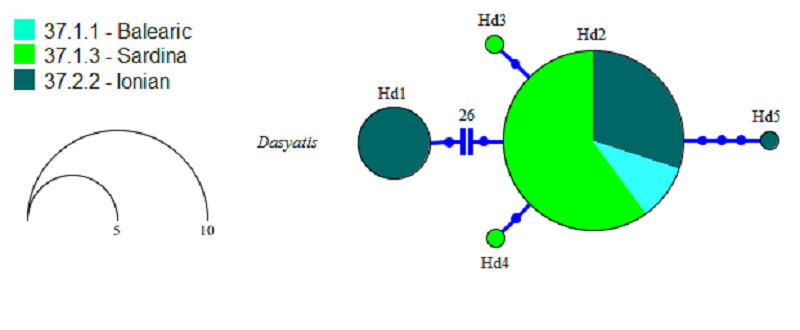

Supplement: S1 Fig — Each circle represents one haplotype and its size is proportional to frequency. Colours indicate the origin of samples according to FAO fishing divisions. (TIF) [file pone.0170244.s005.tif]
